# Magnetic resonance imaging in late pregnancy to improve labour and delivery outcomes – a systematic literature review

**DOI:** 10.1186/s12884-022-05290-x

**Published:** 2022-12-19

**Authors:** Shireen Jaufuraully, Brian Dromey, Lisa Story, Anna L David, George Attilakos, Dimitrios Siassakos

**Affiliations:** 1 Elizabeth Garrett Anderson Institute for Women’s Health, University College London, London, UK; 2 Wellcome / EPSRC Centre for Interventional and Surgical Sciences, University College London, London, UK; 3 Department of Women and Children’s Health, School of Life Course Sciences, King’s College London, London, UK; 4 Fetal Medicine Unit, St Thomas’ Hospital, London, UK; 5 National Institute for Health Research (NIHR) University College London Hospitals Biomedical Research Centre (BRC), 149 Tottenham Court Road, London, UK

**Keywords:** MRI pelvimetry, Cephalopelvic disproportion, Labour, Birth

## Abstract

**Background:**

Magnetic resonance imaging (MRI) provides excellent soft tissue visualisation which may be useful in late pregnancy to predict labour outcome and maternal/neonatal birth trauma.

**Objective:**

To study if MRI in late pregnancy can predict maternal and neonatal outcomes of labour and birth.

**Methods:**

Systematic review of studies that performed MRI in late pregnancy or immediately postpartum. Studies were included if they imaged maternal pelvic or neonatal structures and assessed birth outcome. Meta-analysis was not performed due to the heterogeneity of studies.

**Results:**

Eighteen studies were selected. Twelve studies explored the value of MRI pelvimetry measurement and its utility to predict cephalopelvic disproportion (CPD) and vaginal breech birth. Four explored cervical imaging in predicting time interval to birth. Two imaged women in active labour and assessed mouldability of the fetal skull. No marker of CPD had both high sensitivity and specificity for predicting labour outcome. The fetal pelvic index yielded sensitivities between 59 and 60%, and specificities between 34 to 64%. Similarly, although the sensitivity of the cephalopelvic disproportion index in predicting labour outcome was high (85%), specificity was only 56%. In women with breech presentation, MRI was demonstrated to reduce the rates of emergency caesarean section from 35 to 19%, and allowed better selection of vaginal breech birth. Live birth studies showed that the fetal head undergoes a substantial degree of moulding and deformation during cephalic vaginal birth, which is not considered during pelvimetry. There are conflicting studies on the role of MRI in cervical imaging and predicting time interval to birth.

**Conclusion:**

MRI is a promising imaging modality to assess aspects of CPD, yet no current marker of CPD accurately predicts labour outcome. With advances in MRI, it is hoped that novel methods can be developed to better identify individuals at risk of obstructed or pathological labour. Its role in exploring fetal head moulding as a marker of CPD should be further explored.

**Supplementary Information:**

The online version contains supplementary material available at 10.1186/s12884-022-05290-x.

## Introduction

Although the physiology of labour is generally well understood, and there are known risk factors for operative birth, the ability to predict the mode of birth for any given woman still evades obstetricians. Obstructed labour due to cephalopelvic disproportion (CPD) is not usually diagnosed until labour is established, and is one of the leading indications for caesarean section (CS) [1]. Worldwide, obstructed labour is responsible for up to 3% of maternal and fetal deaths [2] as well as obstetric fistula formation, with two million women living with the condition [3], which has a significant impact upon quality of life. Furthermore, failed instrumental birth and CS at full dilatation increases both maternal and neonatal morbidity and mortality [4], leading to prolonged hospital stays, increased costs to health services, and stress to patients and their families.

In 1948, Mengert stated that there were five components of CPD; namely (1) size and shape of the bony pelvis, (2) size of the fetal head, (3) force exerted by the uterus, (4) mouldability of the fetal head, and (5) presentation and position of the fetus. At the time, only accurate measurements of the first component were possible for pelvimetry [5]. Since then, imaging techniques have progressed significantly. Historically, X ray pelvimetry has been used, but is a source of ionising radiation to the fetus [6] and has not been shown to improve perinatal outcomes or predict labour outcome [7]. Ultrasound (US) in labour is a useful adjunct to digital examination in determining fetal presentation, position, station, and monitoring of labour progress [8]. It has also been proposed to predict the success of operative vaginal birth [9]. In recent years, several studies have explored the relationship between US and magnetic resonance imaging (MRI) findings. MRI appears to be just as accurate as US in determining fetal position and station [10, 11], and is superior to US in predicting neonatal macrosomia [12]. Moreover, MRI can provide substantially more information than US by imaging maternal and fetal bony landmarks and soft tissues. MRI is safe in pregnancy [13] and is increasingly being used in obstetric practice, with numerous studies exploring its role in pelvimetry and predicting labour outcome. The aim of this systematic review is to establish whether MRI conducted in late pregnancy or intrapartum can improve or predict labour outcome, mode of birth, and reduce maternal and neonatal morbidity.

## Methods

The study protocol was registered on PROSPERO international prospective register of systematic reviews (CRD42020220563). The Preferred Reporting Items for Systematic Reviews and Meta-Analyses (PRISMA) guidelines were used to conduct the systematic review.

### Eligibility criteria

All types of study that conducted MRI in the third trimester, labour or immediately postpartum were deemed eligible, including case reports. Studies were included if they imaged any maternal or fetal structures (such as the maternal pelvis) and studied how the findings correlated with birth outcome. Papers were limited to English. Systematic reviews, meta-analyses, narrative reviews, and conference abstracts were excluded.

### Information sources and search strategy

A clinical librarian conducted the systematic search. Web of Science, Cochrane, Embase, and Medline databases were searched with a combination of MESH terms and free text. Duplicates were removed by the clinical librarian and results were emailed to the team. Search terms included: magnetic resonance imaging, pregnancy, vaginal birth, labour, caesarean section, pelvimetry, breech, fetal position, fetal station, and fetal macrosomia. Please see supplementary material (Additional file 1) for the full search strategies.

### Study selection

Two authors (SJ and BD) screened paper titles and abstracts independently. Irrelevant studies were then excluded. Full text articles of selected papers were screened independently. Reference lists of relevant studies were also checked, and studies were selected if they met the selection criteria. Any disagreements were settled by consensus. Full texts were available for all relevant studies. Studies where gestation at birth could not be determined, where imaging took place in early pregnancy, or where mode of birth was not recorded, were excluded. Later studies involving the same patient cohorts that had been previously published by the same authors were also excluded unless different measurements of pelvic/fetal dimensions were performed.

### Data extraction

SJ and BD independently extracted patient data into a standardised Excel spreadsheet. Disagreements were settled by consensus. Study characteristics included study size, study design, and length of gestation at the time the study took place. Mode of birth was recorded, as well as pelvic measurements (such as the fetal pelvic index and measurements of the pelvic inlet), maternal BMI, fetal dimensions, fetal head moulding, and cervical imaging in predicting mode of birth. Where available, the sensitivity and specificity of these were assessed.

### Quality assessment of studies

Study design, type, size, and selection criteria for pregnant women to undergo MRI or MRI with pelvimetry were assessed. Case-control studies, cohort and case reports/series were assessed using the study quality assessment tool provided by the National Institutes of Health. Randomised controlled trials (RCTs) were assessed using the Cochrane Collaboration’s tool for assessing risk of bias.

### Result synthesis

Due to the small number and significant heterogeneity of studies, a meta-analysis was not performed. A narrative synthesis of results has been performed. All tables were made using Microsoft Excel for Mac (Excel Version 16.49).

## Results

### Study selection

**Fig. 1:**
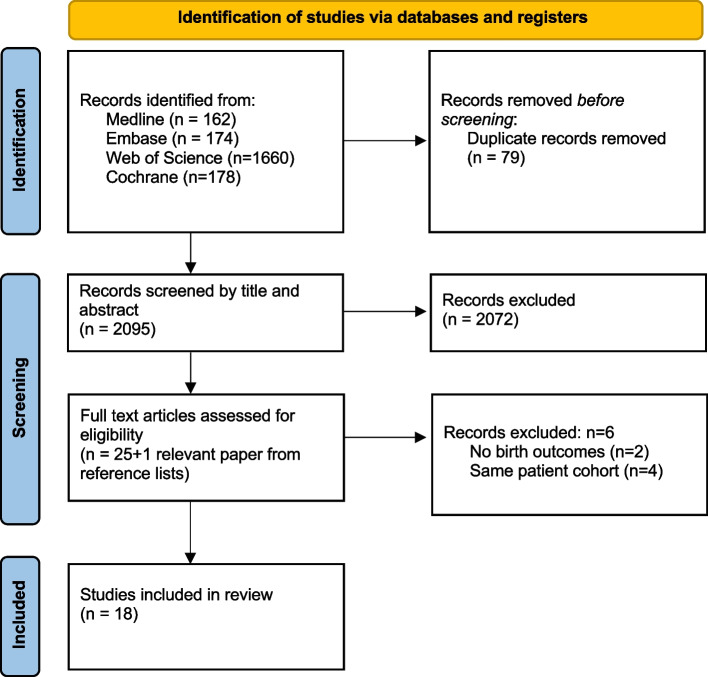
Flow diagram for study selection adapted from PRISMA 2020

The search produced a total of 2174 papers published between 1980 and 2021 (Fig. 1). The clinical librarian removed 79 duplicates. The remaining 2095 studies were screened by title and abstract, and a further 2072 irrelevant studies were excluded. Full texts of the remaining 23 studies were reviewed. 6 studies were excluded for the following reasons: 2 studies gave no details about birth outcomes, and 4 studies reported the same patient cohorts that had been previously published by the same authors. This left 17 papers for review. One relevant paper was extracted from reference lists, leaving 18 papers for analysis.

### Study characteristics

Study characteristics are shown in Table 1. There were 15 cohort studies, one case series, one RCT and one case-control study.

**Table 1:** Study characteristics

First Author/Year	Study design	Size	Timing of MRI	Outcomes assessed
Korhonen U, 2015 [14]	Retrospective cohort	274	10 days prior to birth	FPI in predicting CPD in cephalic presentation
Gleason RL, 2018 [15]	Cohort	287	Beyond 36 weeks	FPI, CPD index, and fetal head volume vs pelvic volume in predicting CPD in cephalic presentation
Sporri S, 2002 [16]	Cohort	38	37 weeks	FPI and CPD index in predicting CPD in cephalic presentation
Fox LK, 2004 [17]	Cohort	16	37–38 weeks	FPI in predicting CPD with previous CS
Sporri S, 1997 [18]	Case-control	41	Postpartum	Fetal head volume vs pelvic volume for predicting CPD
Franz M, 2017 [19]	Retrospective cohort	223	37–38 weeks	Pelvic inlet measurements in women with suspected CPD
Li YG, 2018 [20]	Cohort	244	40 weeks	Pelvimetry in predicting mode of birth in cephalic presentation
Zaretsky AJM, 2005 [21]	Cohort	101	41 weeks	Pelvimetry in predicting mode of birth in cephalic presentation
Hoffman J, 2016 [22]	Retrospective cohort	240	37.5+/−1.6 weeks	Breech presentation and mode of birth
Klemt A-S, 2019 [23]	Cohort	367	39–41 weeks	Breech presentation and mode of birth
Berger R, 1994 [24]	Cohort	33	1–7 days prior to birth	Breech presentation and mode of birth
Van Loon A, 1997 [25]	RCT	235	37 weeks and over	Breech presentation and mode of birth
Bamberg C, 2017 [26]	Case series	1	37 + 5 27	Fetal head moulding
Ami O, 2019 [27]	Cohort	27	37 weeks and over	Fetal head moulding
Sabir N, 2000 [28]	Cohort	21	Prior to induction	Cervical changes
Chan YL, 1998 [29]	Cohort	91	35–41 weeks	Cervical changes
Pates JA, 2007 [30]	Retrospective cohort	93	41 weeks	Cervical changes
Tejada BM, 2011 [31]	Cohort	100	18–34 weeks	Cervical changes

*FPI* Fetal pelvic index

### Quality assessment

The majority of studies were of fair to good quality, with one being of poor quality. The single RCT was at low risk of bias (please refer to supporting information Additional file 2).

### Predictors of labour outcome

#### The fetal-pelvic index

Several studies have explored whether the Fetal Pelvic Index (FPI) (Table 2) can predict labour outcome. It was not a clinically useful tool in predicting labour outcome in women undergoing X Ray or MRI pelvimetry for breech or cephalic presentation with clinical concerns regarding CPD. The FPI had a low area under the curve (AUC) of 0.686, with sensitivity of 0.6 and specificity of 0.34 to predict CPD [14]. This finding has been further confirmed by a feasibility study conducted in Ethiopia, which found the AUC for the FPI to predict CPD was 0.616 [15]. Similarly, Sporri et al. recruited women at high risk for labour dystocia and took MRI measurements of the maternal pelvis (Table 3). Fetal dimensions were taken with US. Sensitivity and specificity for the FPI to predict CPD was 59 and 64% respectively [16].

**Table 2:** The fetal-pelvic index [32] and cephalopelvic disproportion index [33]

**Fetal-Pelvic Index**	
Combines 4 measurements of the fetal head circumference and abdominal circumference with the maternal pelvic inlet and outlet measurements.	
The sum of the two most positive fetal-pelvic circumference differences	
A positive FPI should identify fetuses larger than the maternal pelvis	
A negative FPI should identify fetuses smaller than the maternal pelvis	
Calculation: HC-IC, HC-MC, AC-IC, AC-MC	
**Cephalopelvic Disproportion Index**	
The smallest pelvic diameter (either the anteroposterior of the inlet or the bispinal of the midpelvis) compared to the biparietal diameter of the fetal head	
The difference between the two indicates how much wider the smallest diameter of the bony pelvis is than the fetal skull	
A positive index is present if the pelvic diameter is less than 9 mm wider than the biparietal diameter.	

*HC* Head circumference, *AC* Abdominal circumference, *IC* Pelvic inlet circumference, *MC* Mid-cavity circumference

**Table 3:** Common measurements of the maternal pelvis

Pelvic inlet	Mid pelvis	Pelvic outlet
Obstetric conjugate (measured from the sacral promontory to the upper border of the symphysis pubis)	Sagittal diameter	Sagittal diameter
Transverse diameter	Interspinal diameter	Intertuberous diameter
Circumference	Circumference	

In contrast, a small study concluded that a considerably unfavourable FPI was associated with failed vaginal birth after caesarean (VBAC). Of the 13 patients, 7 had a vaginal birth. Of the six who had a CS, 2 had positive FPIs (both + 0.7) [17].

#### The Cephalo-pelvic disproportion index

In the study mentioned above, Sporri et al. concluded that although the CPD index (Table 2) had high sensitivity (85%), for predicting dystocia and labour outcome, specificity was low (56%) [16]. The AUC for the CPD index was 0.556 in another study, indicating that it is a suboptimal predictor of CPD [15].

#### Comparison of fetal head volume with pelvic volume

This method was developed by Sporri et al. Woman who had CS for suspected CPD and failure to progress underwent postpartum MRI scans. Maternal pelvis capacity was compared with both fetal head volume from antepartum US and postnatal measurement of head volume within 12 hours of birth. 28 women underwent CS for suspected CPD. CPD was defined as head volume exceeding the smallest pelvic inlet or mid pelvis capacity. When US assessed antepartum fetal head volume was related to the smallest pelvic capacity of the inlet or midpelvis, sensitivity for predicting CPD was 89%. Postpartum measurement of fetal head volume in relation to pelvic volume gave a sensitivity of 96% for predicting CPD [18]. This method was further evaluated in a later study by the same authors, with women undergoing antenatal MRI at 37 weeks’ gestation who were deemed at risk of dystocia; sensitivity was 100% but specificity was only 24% [16]. Gleason et al. also found that it was a poor predictor for CPD, with an AUC of 0.571 [15].

#### The pelvic inlet

Obstetric conjugate (OC) measurements have been used to preselect women for trial of vaginal birth. For high-risk patients (breech, suspected CPD, previous pelvic trauma) selected to have a vaginal birth, a larger OC was selected for breech babies compared to those with cephalic presentation (12.7 ± 0.89 cm vs 12.2 ± 0.98). The OC was not significantly different between women who had vaginal birth and emergency CS (12.5 ± 0.9 vs 12.3 ± 1.1 cm). Vaginal birth rates were similar between women with cephalic and breech cases; 70.3 and 75.0% respectively [19]. An OC measurement of 12 cm has been proposed by other authors as a cut off for selection of patients for trial of vaginal breech birth [22, 23]. After doing so, two studies did not find significant differences in the OC between women who had a vaginal birth and an emergency CS [22, 24]. However, other measurements of the midpelvis and pelvic outlet were significantly different between women who had a vaginal birth and an emergency CS, and are discussed in a later section [22]. In contrast, only the OC was significantly larger in women who had a vaginal breech birth in the Frankfurt Breech at Term study. Although the difference was small, the mean OC varied significantly (12.9+/− 0.8 cm) in the vaginal breech birth group versus 12.6+/− 0.8 cm in the CS group. 65.7% of women achieved a successful vaginal birth [23]. One RCT performed MRI pelvimetry on women with breech presentation and used an OC cut-off of 11 cm. A minimum transverse pelvic inlet of 12.5 cm was also required for trial of vaginal birth. Although the overall CS rates were not significantly different between the two groups, MRI significantly reduced the emergency CS rate in the study group (19% vs 35%) but also increased the elective CS rate in the study group [25].

In women with cephalic babies, the pelvic inlet, including the OC, has been demonstrated to be significantly smaller in women who had CS for dystocia compared with those with vaginal births [16, 18, 21]. In one study, the OC measured 10.8+/− 0.9 cm, 11.9+/− 0.9 cm and 11.8+/− 0.7 cm in women who had CS, instrumental birth, and vaginal birth respectively [16]. Similarly, the transverse diameter of the maternal pelvic inlet (134.71 ± 7.53 vs. 131.62 ± 9.16 mm), was significantly larger in women with vaginal birth compared with women who underwent CS for suspected CPD in a study; however, this difference was only 3mm [20].

#### The midpelvis & pelvic outlet

In women undergoing induction of labour (IOL), the mid pelvis anterior-posterior diameter and interspinous diameter (ISD) were significantly smaller in woman who had CS for dystocia compared with those with vaginal births; 116.2+/− 8.0 mm vs 124.6 +/− 8.2 mm and 113.0 +/− 9.7 mm vs 119.1+/− 8.4 mm respectively [21]. This is supported by the study by Sporri et al., where the midpelvis was significantly smaller in women with CS for CPD than those who had vaginal birth [18], and by Li et al., where the posterior sagittal diameter of the midpelvis was larger in women with vaginal birth (45.92 ± 6.71 vs. 42.84 ± 7.53 mm) [20].

In women with breech births, ISD was significantly associated with birth outcome. Vaginal birth success rate was 79% with ISD over 11 cm. However, 58% of patients with an ISD less than 11 cm also had a successful vaginal birth, and would have undergone unnecessary CS. These findings reflect a sensitivity of 71%, a specificity of 53%, an an AUC of 67.7% for predicting successful vaginal birth [22]. Similarly, another study found that women with ISD of less than 11 cm were significantly more likely to undergo CS for failure to progress in breech presentation compared with those who had a successful vaginal birth (10.9+/− 1 vs. 11.6+/− 0.7 cm) [24]

The RCT discussed above proposed midpelvic and pelvic outlet cut-offs for trial of vaginal breech birth [25] (Table 4). With an intertuberous distance of 10.9 cm or less (similar to the 10.5 cm proposed by Van Loon et al. [25]), the FRABAT study found that the emergency CS rate was 100% [22].

**Table 4:** Proposed pelvic midpelvis and pelvic outlet measurements required for trial of breech birth

Midpelvis measurements	Minimum dimensions
Anteroposterior midpelvic distance	≥12·0 cm
Anteroposterior pelvic outlet	≥11·0 cm
Transverse midpelvic distance (interspinal distance)	≥9·5 cm
Transverse pelvic outlet (intertuberous distance)	≥10·5 cm

Adapted from Van Loon et al. [25]

#### Shape of the maternal pelvis

In two studies by Sporri et al., the overall shape of the maternal pelvis was studied as the android and platypelloid were considered abnormal due to the known increased risk of CPD [16]. This was a descriptive diagnosis using the classification from Caldwell and Moloy (Table 5) [18, 34].

**Table 5:** Pelvic shape classifications

Pelvis type	Shape
Anthropoid	Long, narrow, oval
Gynecoid	Round
Platypelloid	Wide or transverse oval appearance
Android	Wedge-shaped or blunt heart-shaped inlet

Adapted from Caldwell and Moloy [34]

Five of 28 women with CPD had either an android or platypelloid pelvis. 50% of women with CPD were diagnosed with having an ‘abnormality of the pelvis’ compared with one woman who had a vaginal birth. Additionally, more cases of malposition were associated with an abnormal shape of the pelvis [18]. The later study also observed that patients with an abnormal pelvis were more likely to have an operative birth compared to a normally shaped pelvis (55% versus 19%) [16].

#### Maternal BMI

Raised BMI could be an indicator for CPD. In the previously mentioned study by Li et al., BMI over 27.6 had an AUC of 0.726 (*p* < 0.001, 95% CI of 0.676–0.791). Sensitivity and specificity were 56.2 and 84.4% respectively for predicting CPD [20]. BMI was also significantly larger in women undergoing CS for CPD compared with women who had a normal vaginal birth in one study [15].

#### Fetal dimensions

Neonatal weight and head circumference are significantly larger in women who have a CS for CPD than those who have a vaginal birth [18, 20]. In a study in China, fetal weight had the biggest AUC in predicting CPD; sensitivity was 96.9% and specificity was 78.4%. Fetal weight less than 3.5 kg was proposed to be an important indicator of successful vaginal birth. Body weight, head circumference and body length were statistically smaller in women who had a vaginal birth compared with those who had a CS for dystocia (head circumference: 35.09 ± 1.28 vs. 32.85 ± 1.16 cm; body length: 51.88 ± 1.16 vs. 50.34 ± 1.33 cm; and body weight: 3.90 ± 0.28 vs. 3.26 ± 0.33 kg) [20]. Although Gleason et al. observed no difference in fetal head or abdominal measurements, they found that ratios of fetal dimensions to maternal inlet and midpelvis were all higher in the CPD compared to the vaginal birth groups [15]. Similarly, although Zaretsky et al. found that no single fetal measurement was statistically associated with dystocia, the ratio of MR fetal head volume to pelvic soft tissue volume is significantly associated with dystocia, with an AUC of 0.64 [21].

In studies of breech babies, women who had CS generally had bigger and longer babies [22]. Both transverse breech diameters (11.7+/− 0.8 vs 10.6+/− 1 cm), estimated fetal weight, and measured weight (3760+/− 370 vs 3080+/− 360 g) were significantly larger in CS for obstructed labour compared with vaginal birth. All babies less than 3.3 kg were born vaginally [24]. Van Loon et al. also observed that mean birthweight was significantly lower in babies born vaginally [25].

### Moulding of the fetal skull

Bamberg et al. captured real time birth in an open MRI in 2012. The woman was 37 + 5 weeks’ pregnant with a singleton cephalic baby. The authors demonstrated fetal head moulding during active second stage by measuring the fronto-occipital diameter (FOD) and distance from the vertex to the base of the fetal skull. In an occiput anterior position, FOD was 10.3 cm. During pushing, the fetal skull became elongated and deformed, with the FOD increasing to 11.2 cm during crowning. The distance from the vertex to base of the fetal skull decreased from 6.4 to 5.6 cm at expulsion [26]. In contrast, Ami et al. demonstrated that the largest change associated with moulding was the reduction of FOD [27]. Seven women were imaged during the second stage of labour. All seven fetuses demonstrated a degree of moulding. Two of three fetuses with the greatest moulding required CS; one for failed forceps and another for lack of engagement of the fetal head. Interestingly, the fetus with the greatest degree of moulding and brain shape deformation was born spontaneously, and weighed 4525 g. It also had low Apgar scores, potentially showing that brain deformation wasn’t tolerated by the fetus. This would also indicate that degree of moulding does not always predict type of birth. The two fetuses with the greatest change in FOD that went on to have CS reinforce the difficulty that can occur when using pelvimetry alone to predict vaginal birth [27].

### Cervical imaging

A study on women undergoing oxytocin IOL found that there was no correlation between cervical signal intensities and failed IOL. Only signal intensities of the posterior cervix showed marginal significance in predicting labour (*p* = 0.567). Although not significant, 85% of patients where subjective visual estimation of signal intensity was high had a vaginal birth. No patients with visually assessed low signal intensity had a vaginal birth. Visual sensitivity and specificity were 84.7 and 100% respectively [28]. In contrast, another study on 91 women undergoing MRI for previous CS found that in the 79 women who laboured spontaneously, there was significant correlation between high signal intensity, relaxation times and external os diameter with interval to birth [29]. However, relaxation time did not correlate with clinical Bishop score or obstetric outcome in women underdoing IOL [30].

MRI has also been used to assess whether changes in cervical stroma differentiation can predict preterm birth. In women with intact membranes, the sensitivity and specificity of low stromal differentiation to predict preterm birth was 23 and 95% respectively. A shorter cervix, already a predictor of preterm birth, was associated with low stromal differentiation. The authors concluded that there is no benefit in MRI over US for cervical length in predicting preterm birth [31].

## Discussion

In this systematic review, we found that no single marker of CPD yields both high sensitivity and specificity in predicting labour outcome. Although individual pelvis dimensions, such as the OC, were significantly different between patients who had CS and vaginal birth, some of these differences were only 3 mm, and raise the question of whether such small differences in pelvic capacity could differentiate between women with CS for CPD and vaginal birth. MRI may allow better selection of women undergoing trial of vaginal breech birth, with proposed OC and intertuberous distance cut-offs of 11-12 cm and 10-11 cm respectively. However, most studies failed to consider fetal breech proportions in relation to maternal pelvic dimensions, potentially excluding women with small OCs who otherwise would have had a successful vaginal breech birth. Moreover, no studies were able to demonstrate which neonates were at risk of head entrapment, so this observation must be looked upon with caution.

Additionally, the literature suggests that high fetal and maternal weight are risk factors for CPD, and maternal soft tissues can play a key role in obstructed labour. Nevertheless, the studies of live birth indicate that pelvimetry is not merely as simple as assessing maternal pelvis capacity in relation to fetal dimensions, as the fetal head can undergo a large degree of moulding and deformation during labour, and women can still have a successful vaginal birth. New methods for assessing pelvic capacity should be developed that also consider the overall shape of the maternal pelvis, as the few studies included suggest that the shape of the pelvis influences the risk of operative birth and CS for CPD.

Although numerous studies have been conducted on cervical imaging, few have explored its use in predicting labour outcome. Those that have are largely conflicting in their findings.

Although MRI can successfully demonstrate cervical anatomy, the included studies question its use when US can demonstrate cervical length (an established marker of threatened preterm labour) and is a cheaper imaging modality [29].

This is the first systematic review to assess the role of MRI in pelvimetry and predicting labour outcome, and encompasses studies from a wide range of countries. As the search was limited to English, some papers could have been missed. Most studies did not have a control group. Additionally, three of the largest studies were retrospective in nature, and therefore at risk of bias. Due to the small number of studies and substantial heterogeneity in MRI indication, and reporting of outcomes, the results are likely not generalisable. At present, the role of MRI in predicting mode of birth and CPD cannot be recommended until new markers of CPD and potential novel imaging techniques are developed.

## Conclusion

MRI may be the best imaging modality to explore all aspects of CPD as described by Mengert et al. Its role in exploring the underlying mechanism of fetal head moulding as a marker of CPD should be further explored. No current markers of CPD accurately predicts labour outcome. MRI may however play a role in better selecting patients for trial of vaginal breech birth. Its use remains uncertain with cervical imaging when US is a cheaper and quicker imaging modality. With advances in MRI, it is anticipated that novel, standardised methods can be developed to better identify individuals at risk of obstructed or pathological labour.

## Supplementary Information

**Additional file 1.**

**Additional file 2.**

## Data Availability

All data generated or analysed during this study are included in this published article]and its supplementary information files].
