# Supplementary material for: Magnetic resonance imaging in late pregnancy to improve labour and delivery outcomes – a systematic literature review
Source: BMC Pregnancy Childbirth. 2022 Dec 19;22:949. doi: 10.1186/s12884-022-05290-x (PMC9761997; doi:10.1186/s12884-022-05290-x)
Supplement: Supplementary file 1 — Additional file 1. [file 12884_2022_5290_MOESM1_ESM.doc]

Magnetic Resonance Imaging in Late Pregnancy to Improve Labour and Delivery Outcomes – A Systematic Literature Review : systematic searches

Database: Embase <1980 to 2020 Week 47>

Search Strategy:

--------------------------------------------------------------------------------

1 exp nuclear magnetic resonance imaging/

2 (MRI* or magnetic resonance imaging).mp.

3 pregnancy/

4 (pregnant or pregnanc*).mp.

5 1 or 2

6 3 or 4

7 5 and 6

8 (birth or twins or multiple pregnanc*).mp.

9 twins/

10 labo?r.mp. or labor/

11 multiple pregnancy/

12 ((Induction adj2 labour) or Antenatal or Intrapartum or Pelvimetry or Breech).mp

13 pelvimetry/

14 breech presentation/

15 (((caesarean section or vaginal birth or vaginal birth) adj3 caesarean section) or Macrosomia).mp.

16 cesarean section/

17 vaginal delivery/

18 macrosomia/ (

19 (Fetal position or fetal weight or fetal or Fetal station or Placenta*).mp.

20 placenta/

21 8 or 9 or 11 or 12 or 13 or 14 or 15 or 16 or 17 or 19 or 20

22 7 and 10 and 21

23 limit 22 to english language

***************************

Database: Ovid MEDLINE(R) and Epub Ahead of Print, In-Process & Other Non-Indexed Citations and Daily <1946 to November 25, 2020>

Search Strategy:

--------------------------------------------------------------------------------

1 exp Magnetic Resonance Imaging/

2 (MRI* or magnetic resonance imaging).mp.

3 Pregnancy/

4 Labor, Induced/ or Labor, Obstetric/

5 Twins/ (15880)

6 exp Multiple Birth Offspring/

7 Breech Presentation/ or Pelvimetry/

8 Vaginal Birth after Cesarean/

9 Fetal Macrosomia/

10 Fetal Weight/

11 Placenta/

12 (pregnanc* or Labo?r or birth* or twin* or multiple pregnanc*).mp.

13 ((Induction adj2 labo?r) or Antenatal or Intrapartum or Pelvimetry or Breech).mp

14 (((caesarean section or vaginal birth or vaginal birth) adj3 caesarean section) or Macrosomia).mp.

15 (Fetal position* or fetal weight or fetal or Fetal station or Placenta*).mp.

16 1 or 2

17 5 or 6 or 7 or 8 or 9 or 10 or 11 or 12 or 13 or 14 or 15

18 3 and 4 and 16 and 17

19 limit 18 to english language

***************************

Web of Science Search Strat and Cochrane Search Strat

|  | (MRI or "magnetic resonance imaging")    *AND*  (Pregnan*)  *AND*  (Labo?r)  *AND*  (birth or twins or "multiple pregnanc*" or "Induction ADJ2 labour" or Antenatal or Intrapartum or Pelvimetry or Breech or "caesarean section" or "vaginal birth" or "vaginal birth ADJ3 caesarean section" or Macrosomia or "Fetal position" or "fetal weight" or fetal or "Fetal station" or Placenta*) |
| --- | --- |
